# Alendronate/cRGD-Decorated Ultrafine Hyaluronate Dot Targeting Bone Metastasis

**DOI:** 10.3390/biomedicines8110492

**Published:** 2020-11-11

**Authors:** Eunsol Lee, Jaeduk Park, Yu Seok Youn, Kyung Taek Oh, Dongin Kim, Eun Seong Lee

**Affiliations:** 1 Department of Biotechnology, The Catholic University of Korea, 43 Jibong-ro, Bucheon-si, Gyeonggi-do 14662, Korea; eunsollee13@gmail.com (E.L.); jduck0309@naver.com (J.P.); 2 School of Pharmacy, SungKyunKwan University, 2066 Seobu-ro, Jangan-gu, Suwon, Gyeonggi-do 16419, Korea; ysyoun@skku.edu; 3 College of Pharmacy, Chung-Ang University, 221 Heukseok dong, Dongjak-gu, Seoul 06974, Korea; kyungoh@cau.ac.kr; 4 Department of Pharmaceutical Sciences, College of Pharmacy, University of Oklahoma Health Sciences Center, 1110 N Stonewall Ave, Oklahoma City, OK 73117, USA; Dongin-Kim@ouhsc.edu; 5 Department of Biomedical Chemical Engineering, The Catholic University of Korea, 43 Jibong-ro, Bucheon-si, Gyeonggi-do 14662, Korea

**Keywords:** hyaluronate dot, alendronate, cyclic RGD, bone metastasis, photodynamic tumor therpy

## Abstract

In this study, we report the hyaluronate dot (dHA) with multiligand targeting ability and a photosensitizing antitumor model drug for treating metastatic bone tumors. Here, the dHA was chemically conjugated with alendronate (ALN, as a specific ligand to bone), cyclic arginine-glycine-aspartic acid (cRGD, as a specific ligand to tumor integrin α_v_β_3_), and photosensitizing chlorin e6 (Ce6, for photodynamic tumor therapy), denoted as (ALN/cRGD)@dHA-Ce6. These dots thus prepared (≈10 nm in diameter) enabled extensive cellular interactions such as hyaluronate (HA)-mediated CD44 receptor binding, ALN-mediated bone targeting, and cRGD-mediated tumor integrin α_v_β_3_ binding, thus improving their tumor targeting efficiency, especially for metastasized MDA-MB-231 tumors. As a result, these dots improved the tumor targeting efficiency and tumor cell permeability in a metastatic in vivo tumor model. Indeed, we demonstrated that (ALN/cRGD)@dHA-Ce6 considerably increased photodynamic tumor ablation, the extent of which is superior to that of the tumor ablation of dot systems with single or double ligands. These results indicate that dHA with multiligand can provide an effective treatment strategy for metastatic bone tumors.

## 1. Introduction

Extremely small-sized particles with a few nanometers in diameter have been extensively utilized to control or visualize biological functions in the field of biology and improve the disease-targeting ability of drugs in the field of pharmaceutics [1,2,3,4]. Indeed, these particles such as quantum dots, carbon dots, and polymeric dots, can efficiently interact with the cell receptors, cell proteins, genes, and cytokines owing to their surface functionality and tailor-made extremely small-sized configuration [5,6,7,8,9]. However, the innate toxicity of such dots should be considered in the course of accompanying various biomedical applications [10]. Especially, the fabrication of biofunctional dots with low cytotoxicity can offer various possibilities for realizing successful dot-based therapeutics and diagnosis [11,12,13].

Recently, our groups developed organic hyaluronate dots with biocompatible/biodegradable properties [4,11,14]. These dots (3–10 nm in diameter) conjugated with an antitumor model drug have exhibited increased in vitro cellular interaction and in vivo tumor targeting ability, resulting in a significant improvement of in vitro and in vivo tumor ablation [4,14]. Based on these studies, we here designed multifunctional hyaluronate dots with more tumor-specific targeting ligands to aggressively attack tumor cells with bone metastasis phenotype, and use them to improve the treatment efficacy of metastatic tumors.

**Figure 1:**
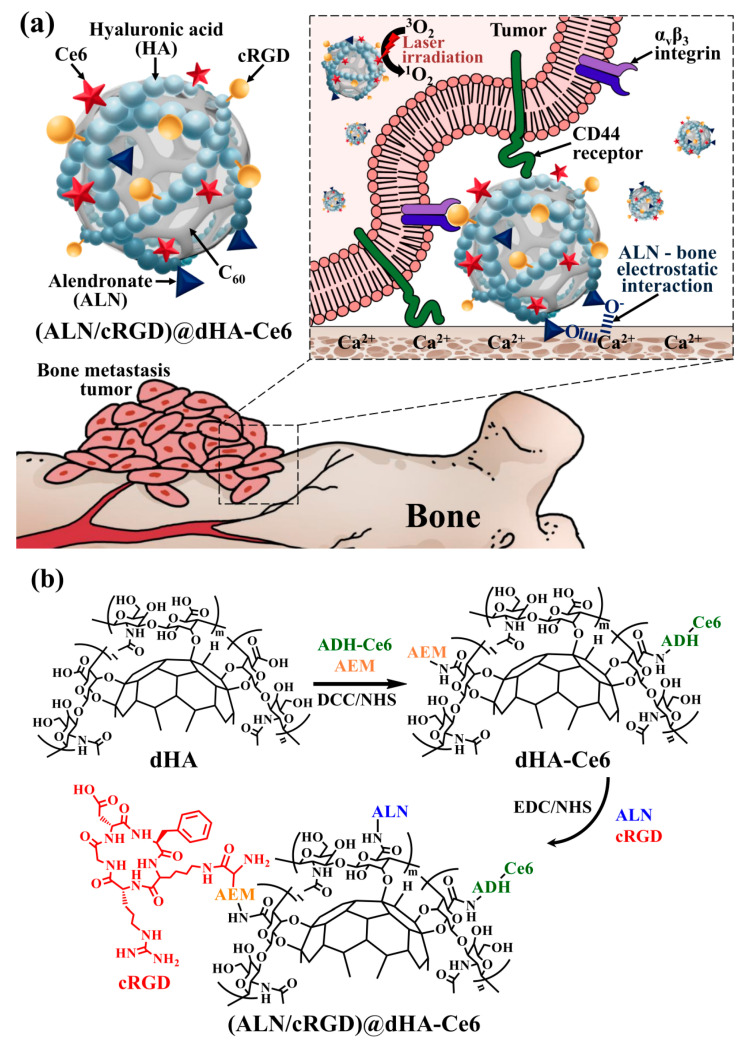
(**a**) Schematic illustration of (ALN/cRGD)@dHA-Ce6. (**b**) Synthesis scheme of the (ALN/cRGD)@dHA-Ce6.

It is known that bone metastasis originating from the relocation of circulating tumor cells usually causes pathogenic infiltration and multiple fractions, which create complicated in vivo conditions [15,16,17,18]. Actually, conventional antitumor drugs are significantly limited in penetration into bone-metastasized tumor tissues owing to the conventional densely packed metastatic tumor tissues in bone regions [19,20,21,22]. However, it is interesting to note that bisphosphonate drugs inducing apoptosis in osteoclasts and inhibiting osteolysis have shown their potential in high interaction with bone tissues owing to their high affinity (e.g., electrostatic interaction) for bone hydroxyapatite, revealing their attractive role as bone-specific ligands [23,24,25,26,27]. Furthermore, it is also known that hyaluronic acid (HA), a linear biodegradable polysaccharide, can specifically interact with CD44 receptors overexpressed on various metastatic tumors [28,29,30,31,32]. The tumor-targeting peptide, cyclic arginine-glycine-aspartic acid (cRGD) can also bind to α_v_β_3_ integrin receptors overexpressed on metastatic breast tumors [33,34,35,36]. Therefore, in this study, the hyaluronate dot (dHA) with multiligand targeting abilities was developed to treat in vivo metastatic bone tumors. First, dHA was synthesized using the chemical conjugating method by using C_60_ and HA [4,14]. Then, the dHAs were chemically conjugated with alendronate (ALN), cRGD, and chlorin e6 (Ce6, as a photodynamic antitumor agent), denoted as (ALN/cRGD)@dHA-Ce6. We expect that the dHA with multiple ligands (HA, ALN, and cRGD) facilitates multiple receptor-mediated cellular internalizations and provides multiple routes for efficient drug uptake in bone-metastasized tumors, resulting in improved photodynamic tumor ablation (Figure 1a). In particular, we investigated the in vitro/in vivo tumor targeting ability and antitumor efficacy of (ALN/cRGD)@dHA-Ce6 against MDA-MB-231 bone-metastasized tumors.

## 2. Materials and Methods

### 2.1. Materials

Hyaluronic acid (HA, M_n_ = 3.7 kDa), dimethyl sulfoxide (DMSO), toluene, *N,N′*-dicyclohexylcarbodiimide (DCC), *N*-hydroxysuccinimide (NHS), *N*-(2-aminoethylmaleimide) (AEM), triethylamine (TEA), adipic acid dihydrazide (ADH), alendronate sodium salt (ALN), *N*-(3-dimethylaminopropyl)-*N*′-ethylcarbodiimide hydrochloride (EDC), deoxychlolic acid (DOCA), 4-dimethylaminopyridine (DMAP), pyridine, 9,10-dimethylanthracene (DMAT), hydroxyapatite, sodium hydroxide (NaOH), 4′,6-diamidino-2-phenylindole dihydrochloride (DAPI), formaldehyde, ethanol, and hexamethyldisilazane (HMDS) were purchased from Sigma-Aldrich (St. Louis, MO, USA). C_60_ was purchased from NanoLab Inc. (Waltham, MA, USA). Chlorin e6 (Ce6) was purchased from Frontier Scientific Inc. (Logan, UT, USA). cRGD [cyclo(Cys-Lys-Arg-Gly-Asp-D-Phe)] was purchased from Peptron Inc. (Daejeon, Korea). Penicillin, streptomycin, fetal bovine serum (FBS), RPMI-1640 medium, bovine calf serum (BCS), Dulbecco’s modified eagle medium (DMEM), trypsin, and ethylene diamine tetra-acetic acid (EDTA) were purchased from Welgene Inc. (Seoul, Korea). Type I collagen solution (3 mg/mL, Bovine) was purchased from Advanced BioMatrix (San Diego, CA, USA). Cell Counting Kit-8 (CCK-8) was purchased from Dojindo Molecular Technologies Inc. (Rockville, MD, USA). Wheat Germ Agglutinin Alexa Fluor^®^ 488 conjugate (WGA-Alexa Fluor^®^ 488) was purchased from Life Technologies (Carlsbad, CA, USA).

### 2.2. Synthesis of Hyaluronate Dots (dHA) with ALN, cRGD, and Ce6

Four types of hyaluronate dot (dHA) derivatives were prepared as follows: (1) dHA with Ce6 (dHA-Ce6), (2) dHA with ALN and Ce6 [(ALN)@dHA-Ce6], (3) dHA with cRGD and Ce6 [(cRGD)@dHA-Ce6], and (4) dHA with ALN, cRGD, and Ce6 [(ALN/cRGD)@dHA-Ce6]. First, the dHA (400 mg, prepared after conjugating HA to all π–π carbon bonds of C_60_) was preactivated in DMSO (15 mL) containing DCC (20 mg), NHS (25 mg), AEM (8 mg), and TEA (1 mL) for 1 day and reacted with preactivated Ce6 [150 mg, preactivated for 4 h in DMSO (15 mL) containing ADH (35 mg), DCC (20 mg), NHS (25 mg), and TEA (0.5 mL)] for 3 days at 25 °C. The resulting solution was dialyzed using a pre-swollen dialysis membrane (Spectra/Por^®^ MWCO 10 kDa; Spectrum Lab., Rancho Dominguez, CA, USA) against fresh DMSO for 2 days and then deionized water for 2 days to remove non-reacted chemicals. The dialyzed solution was freeze-dried to obtain dHA-Ce6 [4,14,35,37]. The resulting dHA-Ce6 (200 mg) was reacted with ALN (60 mg) in deionized water (20 mL) containing EDC (40 mg), NHS (45 mg), and TEA (1 mL) for 3 days at 25 °C. The solution was dialyzed against deionized water for 3 days and freeze-dried to obtain (ALN)@dHA-Ce6. Next, to synthesize the (cRGD)@HA-Ce6, the dHA-Ce6 (200 mg) was reacted with cRGD (4 mg) in deionized water (20 mL) for 3 days at 25 °C. The solution was dialyzed and freeze-dried to obtain (cRGD)@dHA-Ce6 [4,35]. Finally, to synthesize (ALN/cRGD)@dHA-Ce6, the dHA-Ce6 (200 mg) was reacted with ALN (60 mg) and cRGD (4 mg) in deionized water (20 mL) containing EDC (40 mg), NHS (45 mg), and TEA (1 mL) for 3 days at 25 °C, and then the resulting solution was dialyzed and freeze-dried.

Additionally, HA grafted with DOCA, ALN, cRGD, and Ce6 was utilized as a control nanoparticle (NP) group, denoted as (ALN/cRGD)@HDOC-Ce6 NP. Here, HA (200 mg) was reacted with DOCA (655 mg) in DMSO (10 mL) containing DCC (345 mg), DMAP (205 mg), and pyridine (0.5 mL) for 2 days at 25 °C. After reaction, the solution was dialyzed using a pre-swollen dialysis membrane (Spectra/Por^®^ MWCO 1 kDa) against fresh DMSO for 2 days and then deionized water for 2 days. The dialyzed solution was freeze-dried to obtain HDOC [37,38]. The HDOC (1.2 g) was again preactivated in DMSO (15 mL) containing DCC (60 mg), NHS (75 mg), AEM (10 mg), and TEA (1 mL) for 1 day and reacted with preactivated Ce6 (225 mg, preactivated for 4 h in DMSO (15 mL) containing ADH (35 mg), DCC (20 mg), NHS (25 mg), and TEA (0.5 mL)) for 3 days at 25 °C. The resulting solution was dialyzed and freeze-dried to obtain HDOC-Ce6 [37,38]. Then, HDOC-Ce6 was reacted with ALN (80 mg) and cRGD (4 mg) in deionized water (30 mL) containing DCC (60 mg), NHS (75 mg), and TEA (1 mL) for 3 days at 25 °C. The solution was dialyzed against deionized water for 3 days and freeze-dried, finally producing (ALN/cRGD)@HDOC-Ce6. Furthermore, the (ALN/cRGD)@HDOC-Ce6 was dissolved in DMSO and dialyzed against fresh phosphate buffered saline (PBS, pH 7.4, 150 mM) for 1 day to prepare (ALN/cRGD)@HDOC-Ce6 NP [39,40].

### 2.3. Characterization of dHA Samples

The chemical structure of each dHA sample and (ALN/cRGD)@HDOC-Ce6 (as a control) was analyzed using a 500 MHz NMR Spectrometer (Bruker, Billerica, MA, USA) [4,14,35,37]. The particle size and morphology of each dHA sample and (ALN/cRGD)@HDOC-Ce6 was analyzed using a transmission electron microscope (TEM, Carl zeiss, Oberkochen, Germany) [4,14,35,37]. In addition, the particle size and zeta potential of each dHA sample and (ALN/cRGD)@HDOC-Ce6 NP (0.1 mg/mL) in PBS (pH 7.4, 150 mM) was measured using Zetasizer 3000 (Malvern instruments, Malvern, UK) [4,14,35,37].

The light emission spectrum (at λ_ex_ of 400 nm and λ_em_ of 600–750 nm) of each dHA sample with fluorescent Ce6 (equivalent Ce6 10 μg/mL) or (ALN/cRGD)@HDOC-Ce6 NP with fluorescent Ce6 (equivalent Ce6 10 μg/mL) in PBS (pH 7.4, 150 mM) was measured using a RF-5301PC spectrofluorophotometer (Shimadzu, Kyoto, Japan) [14,41,42].

The generation of singlet oxygen by each dHA sample (equivalent Ce6 10 μg/mL), (ALN/cRGD)@HDOC-Ce6 NP (equivalent Ce6 10 μg/mL), and free Ce6 (10 μg/mL) in PBS (pH 7.4, 150 mM) was confirmed using a RF-5301PC spectrofluorophotometer by analyzing the fluorescence of 9,10-dimethylathracene (DMAT, at λ_ex_ of 360 nm and λ_em_ of 380-550 nm) [14,35,37,41,42,43,44]. Briefly, each sample (in pH 7.4, 150 mM PBS) was mixed with DMAT (20 mmol) and irradiated using a 670 nm laser source (5.2 mW/cm^2^ for 10 min). After 1 h, when the DMAT fluorescence intensity reached a plateau, the change in the fluorescence of DMAT (F_f_–F_s_, F_f_ is the fluorescence intensity of pure DMAT and F_s_ is the fluorescence intensity of DMAT mixed with the sample) was plotted. Here, the fluorescence of DMAT decreases because DMAT selectively captures singlet oxygen molecule [14,35,36,41,42,43,44].

### 2.4. Hydroxyapatite Binding Analysis

The binding affinity of each dHA sample (1 mg/mL) and (ALN/cRGD)@HDOC-Ce6 NP (1 mg/mL) to bone-like hydroxyapatite particles (10 mg) in PBS solution was analyzed using a Cary 1E UV/visible spectrophotometer (Varian Inc., Palo Alto, CA, USA) by analyzing the light absorbance of solution [41,44,45]. Here, each sample (in pH 7.4 150 mM PBS) was mixed with hydroxyapatite particles (≈2.5 μm in diameter) and incubated with mechanical shaking (100 rpm) at 37 °C. At the specified time point, the incubated sample was centrifuged at 5000 rpm for 5 min, and the supernatant was collected. The light absorbance of the collected supernatant was measured at a wavelength of 670 nm. Consequently, the binding affinity was calculated by the following formula: binding affinity (%) = (A_0_ − A)/A_0_ × 100 (%), where A_0_ is initial absorbance and A is absorbance at the specified time [46].

### 2.5. Cell Culture

Human breast carcinoma MDA-MB-231 cells, human lung carcinoma A549 cells, human breast carcinoma BT-474 cells, and mouse embryo fibroblast NIH3T3 cells were purchased from the Korean Cell Line Bank. MDA-MB-231, A549, and BT-474 cells were cultured in the RPMI-1640 medium containing 1% penicillin-streptomycin and 10% FBS in 5% CO_2_ atmosphere at 37 °C. NIH3T3 cells were cultured in the DMEM medium containing 1% penicillin-streptomycin and 10% BCS in 5% CO_2_ atmosphere at 37 °C. Prior to testing, the cells (1 × 10^6^ cells/mL) were harvested by trypsinization using 0.25% (wt./vol.) trypsin/0.03% (wt./vol.) EDTA solution and seeded in well plates containing the RPMI-1640 or DMEM medium [4,11,14].

### 2.6. In Vitro Phototoxicity

MDA-MB-231, A549, BT-474, and NIH3T3 cells were used to verify the phototoxicity of each dHA sample and (ALN/cRGD)@HDOC-Ce6 NP under light irradiation. Here, the cells were incubated in type I collagen solution without or with hydroxyapatite particles (mimicking the live in vivo bone environment) and then incubated at 37 °C for 2 h. Next, the collagen gel containing cells in RPMI-1640 or DMEM medium was flipped to expose the cells on the surface as shown in Figure 4 of [46]. These cells were incubated with each dHA sample (equivalent Ce6 10 μg/mL), (ALN/cRGD)@HDOC-Ce6 NP (equivalent Ce6 10 μg/mL), and free Ce6 (10 μg/mL) at 37 °C for 4 h, washed three times with PBS (pH 7.4, 150 mM), and then were irradiated using a 670 nm laser source (5.2 mW/cm^2^ for 10 min). The treated cells were further incubated at 37 °C for 12 h. Subsequently, we measured cell viability using a CCK-8 assay [14,35,36,41,42]. In addition, the original toxicity of each dHA samples and (ALN/cRGD)@HDOC-Ce6 NP without light irradiation was evaluated after 24 h of treatment [14,35,36,41,42].

### 2.7. In Vitro Cellular Uptake

MDA-MB-231 cells were incubated with each dHA sample (equivalent Ce6 10 μg/mL), (ALN/cRGD)@HDOC-Ce6 NP (equivalent Ce6 10 μg/mL), and free Ce6 (10 μg/mL) at 37 °C for 4 h. After washing the cells using PBS (pH 7.4, 150 mM), the fluorescence intensity of the cells was analyzed using a FACSCaliburTM flow cytometer (FACSCanto II, Becton Dickinson, Franklin lakes, NJ, USA) [4,14,41,42]. In addition, to visualize the cellular uptake of each sample, the MDA-MB-231 cells were incubated with the each sample for 4 h and then stained using DAPI and WGA-Alexa Fluor^®^488. The stained cells were fixed using using 3.7% formaldehyde in PBS and analyzed using a confocal laser scanning microscope (LSM710, Carl Zeiss, Oberkochen, Germany) [4,14,41,42].

### 2.8. Animal Care

All animal experiments were performed using 6–8 weeks old female BALB/c nude mice (Orient Bio Inc., Seongnam, Korea) and progressed under the guidelines of an approved protocol from the Institutional Animal Care and Use Committee (IACUC, the project identification code: 2018-016, July, 09, 2018) of the Catholic University of Korea [4,41,42].

### 2.9. Ex Vivo Photodynamic Tumor Therapy Using a Bone Metastasis Model

To evaluate the photodynamic antitumor efficacy of each sample using a bone metastasis model, BALB/c nude mice were euthanized using carbon dioxide asphyxiation, and their tibias were extracted under sterile conditions. The separated tibias were placed into 6-well plates and incubated with MDA-MB-231 tumor cells (1 × 10^6^ cells/mL) in RPMI-1640 medium at 37 °C for 48 h [27,47]. The tibias with MDA-MB-231 tumor cells were incubated with each dHA sample (equivalent Ce6 10 μg/mL), (ALN/cRGD)@HDOC-Ce6 NP (equivalent Ce6 10 μg/mL), and free Ce6 (10 μg/mL) at 37 °C for 4 h, washed three times with PBS (pH 7.4, 150 mM), and then irradiated using a 670 nm laser source (5.2 mW/cm^2^ for 10 min). The treated cells on tibias were further incubated at 37 °C for 12 h and then fixed using glutaraldehyde, ethanol, and HMDS. The resulting tibias were monitored using a scanning electron microscopy (SEM, S-4800, Hitachi, Tokyo, Japan) [27,47].

### 2.10. In Vivo Biodistribution

We prepared in vivo bone metastasized tumor model using BALB/c nude mice intraosseously injected with MDA-MB-231 tumor cells (1 × 10^7^ cells in pH 7.4, 150 mM PBS) [48]. After 21 days, the in vivo transplanted tumors were confirmed using a micro CT imaging scanner (CLS140083, PerkinElmer Inc., Waltham, MA, USA) with parameters of 90 kV, 88 μAs, and 4 min scan time [41,42]. Next, each dHA sample (equivalent Ce6 2.5 mg/kg) and free Ce6 (2.5 mg/kg) were intravenously administered to the BALB/c nude mice to treat the MDA-MB-231 metastasized tumor. Importantly, fluorescence images of each sample (with fluorescent Ce6) at the tumor site were obtained at 1, 4, and 8 h post-injection using a Fluorescence-labeled Organism Bioimaging Instrument (FOBI, Neo-Science, Seoul, Korea) [4,49]. In addition, the tumors (in the right leg) and major organs (heart, lung, liver, kidney, and spleen) were harvested from the BALB/c nude mice (at 24 h post-injection) euthanized by carbon dioxide asphyxiation, and then assessed using FOBI analysis [4,49].

### 2.11. In Vivo Photodynamic Tumor Therapy

Each dHA sample (equivalent Ce6 2.5 mg/kg), free Ce6 (2.5 mg/kg), and control (saline) was intravenously injected into the tumor-bearing BALB/c nude mice through their tail vein. At 12 h post-injection, the metastasized tumor sites (tibias) of the BALB/c nude mice were locally irradiated for 40 min at a light intensity of 5.2 mW/cm^2^ with a 670 nm laser source. The tumor volume was calculated using the following formula: tumor volume = length × (width)^2^/2. The relative tumor volume change (V/V_0_), where V is the tumor volume at a given time and V_0_ is the initial tumor volume, was plotted to evaluate the photodynamic tumor ablation of (ALN/cRGD)@dHA-Ce6 [14,35,36]. Furthermore, the micro CT images of tumor sites at 14 days post-injection were captured using a micro CT imaging scanner [41,42].

### 2.12. Statistics

All of the experimental results were analyzed using Student’s *t*-test or ANOVA at a significance level of *p* < 0.01 (**) [4,14,35].

## 3. Results and Discussion

### 3.1. Synthesis of dHA Samples

To fabricate the hyaluronate dot (dHA) with multiligand targeting ability to treat bone metastatic tumors (Figure 1a), we first synthesized dHA as a backbone carrier; the dHA was prepared by conjugating the hydroxy groups of HA to all π–π carbon bonds of C_60_ [4,14]_._ The obtained dHA (M_w_ = 13.0 kDa) was coupled with a photodynamic antitumor agent Ce6 (preactivated using ADH) using DCC, NHS, AEM, and TEA in DMSO solvent and then chemically linked with ALN (for targeting Ca^2+^ in bone) and cRGD (for targeting integrin α_v_β_3_) using EDC, NHS, and TEA in DMSO solvent as shown in Figure 1b [4,14,35,36]. As a result, we fabricated the (ALN/cRGD)@dHA-Ce6 and evaluated the conjugation molar ratio of Ce6, ALN, and cRGD to dHA using proton nuclear magnetic resonance (^1^H-NMR) (Figure S1). The ^1^H-NMR results show that the conjugation molar ratio of Ce6, ALN, and cRGD in (ALN/cRGD)@dHA-Ce6 were 0.14, 0.43, and 0.10, respectively, per one repeating unit of HA, estimated after analyzing the integration ratio of the peaks from δ 1.21 ppm (-CH from dHA), δ 6.42 ppm (-CH_2_- from Ce6), δ 2.98 ppm (-CH_2_- from ALN), and δ 7.81 ppm (-CH from cRGD). Additionally, (ALN/cRGD)@HDOC-Ce6 (as a control NP with 100 nm in size) was prepared using HA, DOCA, Ce6, ALN, and cRGD (Figure S2). Subsequently, (ALN/cRGD)@HDOC-Ce6 NPs were prepared through the self-assembly process of (ALN/cRGD)@HDOC-Ce6 in PBS (pH 7.4, 150 mM) [39,40].

### 3.2. Characterization of dHA Samples

**Figure 2:**
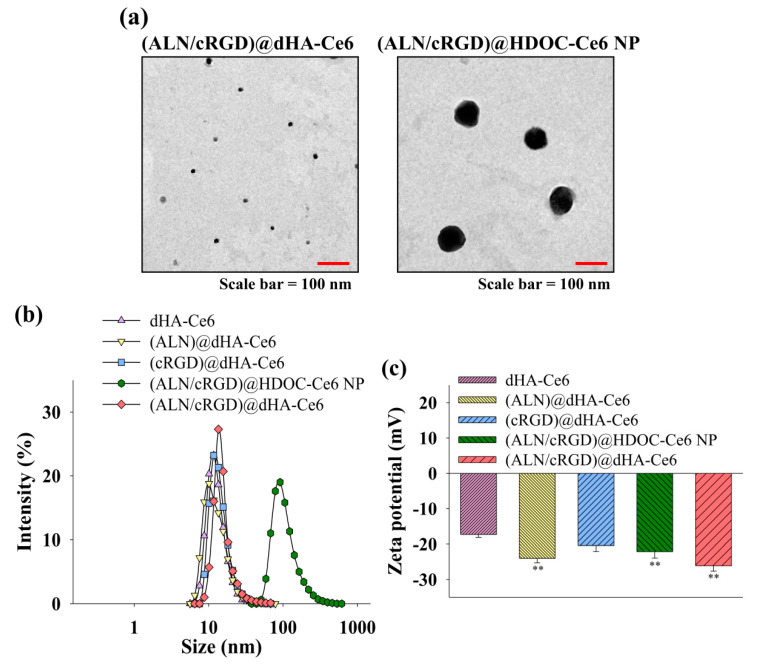

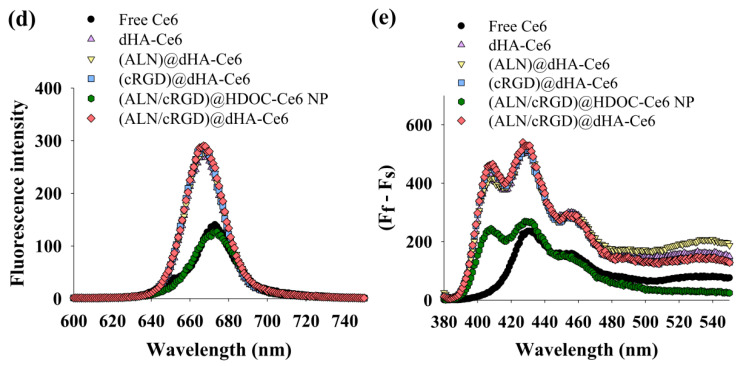
(**a**) TEM images of (ALN/cRGD)@dHA-Ce6 (left) and (ALN/cRGD)@HDOC-Ce6 NP (right). (**b**) Particle size distribution and (**c**) zeta potential values of dHA-Ce6, (ALN)@dHA-Ce6, (cRGD)@dHA-Ce6, (ALN/cRGD)@HDOC-Ce6 NP, and (ALN/cRGD)@dHA-Ce6 at pH 7.4 (n = 3, as multiple experiments, ** *p* < 0.01 compared to dHA-Ce6). (**d**) The emission spectra (at λ_ex_ of 400 nm and λ_em_ of 600–750 nm) of free Ce6 (10 μg/mL) and each sample (equivalent Ce6 10 μg/mL) in PBS (pH 7.4, 150 mM). (**e**) The 9,10-dimethylanthracene (DMAT) fluorescence change (at λ_ex_ of 360 nm and λ_em_ of 380–550 nm) of free Ce6 (10 μg/mL) and each sample (equivalent Ce6 10 μg/mL) in PBS (pH 7.4, 150 mM). The singlet oxygen generation is indicated by the DMAT fluorescence intensity change (F_f_ − F_s_, where F_f_ is the fluorescence intensity of DMAT and F_s_ is the fluorescence intensity of DMAT mixed with the sample).

Figure 2a shows the images of (ALN/cRGD)@dHA-Ce6 and (ALN/cRGD)@HDOC-Ce6 NP obtained using a TEM. TEM images reveal that all dHA samples have an extremely small-sized dot shape with an average diameter of 5–10 nm. In addition, (ALN/cRGD)@HDOC-Ce6 NPs have an average diameter of 100 nm and exhibit almost spherical morphologies. Figure 2b also shows that the average particle sizes of each dHA sample and (ALN/cRGD)@HDOC-Ce6 NP (measured using a Zetasizer 3000) were approximately 10 and 100 nm, respectively.

Figure 2c shows that the zeta potential of all samples was negative at pH 7.4, owing to the presence of the negatively charged HA and Ce6 [50,51]. Especially, (ALN)@dHA-Ce6, (ALN/cRGD)@HDOC-Ce6 NP, and (ALN/cRGD)@dHA-Ce6 have a very reduced zeta potential value owing to the presence of rich phosphoryl groups of ALN [52,53].

Figure 2d shows the fluorescence Ce6 intensity of each dHA sample and (ALN/cRGD)@HDOC-Ce6 NP. Interestingly, free Ce6 or (ALN/cRGD)@HDOC-Ce6 NP exhibited reduced NIR fluorescence intensities at 670 nm, probably owing to the auto-quenching event of Ce6 molecules originated from their Ce6 aggregates formation in aqueous solution [42,45]. However, water-soluble dHA-Ce6, (ALN)@dHA-Ce6, (cRGD)@dHA-Ce6, and (ALN/cRGD)@dHA-Ce6 exhibited increased fluorescence intensity owing to the free mobility of fluorescent Ce6 molecules attached to water-soluble dHA.

Next, the singlet oxygen generation of (ALN/cRGD)@dHA-Ce6 was determined using fluorescent 9,10-dimethylanthracene (DMAT), as shown in Figure 2e. First, all dHA samples highly generated singlet oxygen under light irradiation. However, free Ce6 or (ALN/cRGD)@HDOC-Ce6 NP reduced singlet oxygen generation, owing to the auto-quenching event of aggregated Ce6 molecules [43,54]. These results demonstrate that, unlike (ALN/cRGD)@HDOC-Ce6 NPs, dHA samples can enhance singlet oxygen-mediated phototoxicity to tumor cells during tumor therapy.

### 3.3. Hydroxyapatite Binding Test of dHA Samples

**Figure 3:**
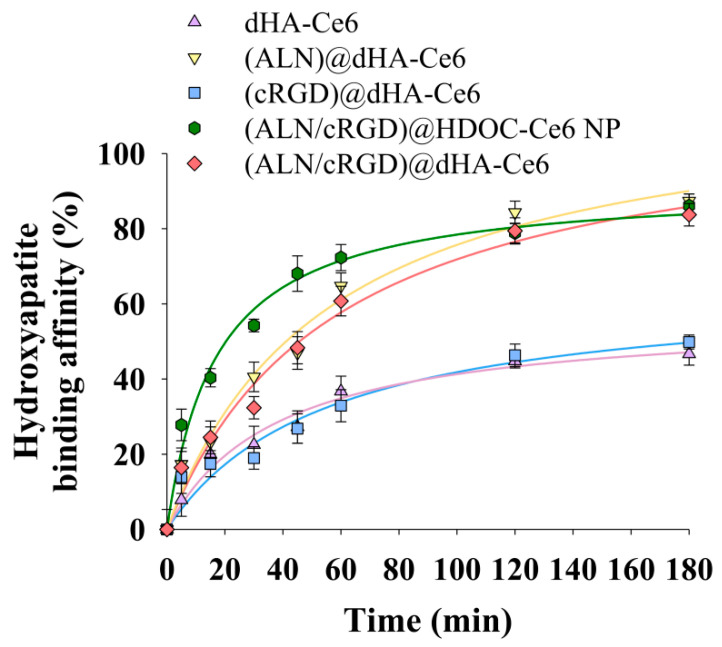
Electrostatic binding affinity of each sample with hydroxyapatite particles (as an in vitro bone model, ≈2.5 μm in diameter) in PBS (pH 7.4, 150 mM) (n = 3, as multiple experiments). Here, dHA-Ce6 (the conjugation molar ratio of Ce6 to one repeating unit of HA was 0.14), (ALN)@dHA-Ce6 (the conjugation molar ratio of Ce6 and ALN to one repeating unit of HA was 0.14 and 0.43, respectively), (cRGD)@dHA-Ce6 (the conjugation molar ratio of Ce6 and cRGD to one repeating unit of HA was 0.14 and 0.10, respectively), (ALN/cRGD)@HDOC-Ce6 NP (the conjugation molar ratio of Ce6, ALN, cRGD to one repeating unit of HA was 0.25, 0.40, and 0.13, respectively), and (ALN/cRGD)@dHA-Ce6 (the conjugation molar ratio of Ce6, ALN, and cRGD to one repeating unit of HA was 0.14, 0.43, and 0.10, respectively) were used as experimental samples (Figures S1–S4).

Figure 3 shows the hydroxyapatite binding affinity of each dHA sample and (ALN/cRGD)@HDOC-Ce6 NP. First, dHA-Ce6 and (cRGD)@dHA-Ce6 without ALN showed a relatively low 35–40 wt.% nonspecific binding affinity after 180 min, while (ALN)@dHA-Ce6, (ALN/cRGD)@HDOC-Ce6 NP, and (ALN/cRGD)@dHA-Ce6 showed increased binding affinity equivalent to approximately 80 wt.%, probably owing to Ca^2+^ (bone)-mediated electrostatic interaction with ALN [25,26,27]. These results indicate that dHA samples with ALN have enhanced bone-targeting ability.

### 3.4. In Vitro Phototoxicity of dHA Samples

**Figure 4:**
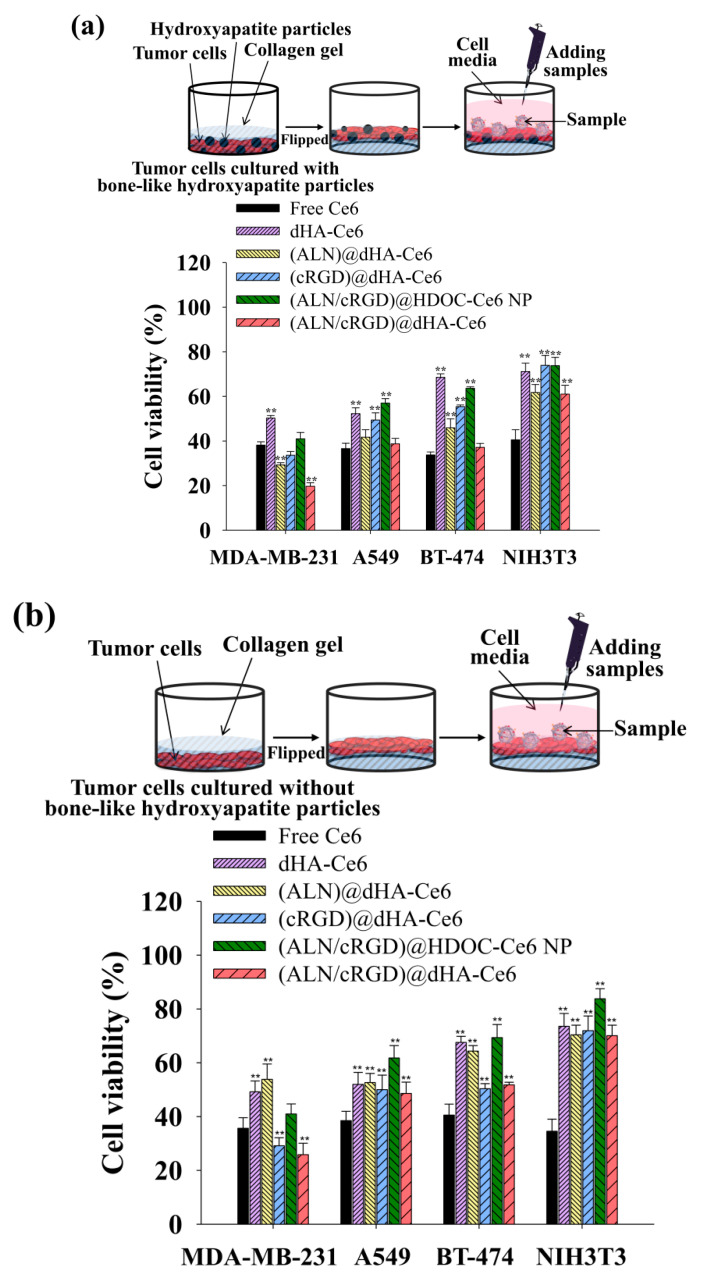
Phototoxicities were determined by a CCK-8 assay of MDA-MB-231, A549, BT-474, and NIH3T3 cells cultured in collagen gel-containing cell medium (**a**) with or (**b**) without hydroxyapatite particles. All cells were treated with free Ce6 (10 μg/mL) or each sample (equivalent Ce6 10 μg/mL) for 4 h and illuminated for 10 min at a light intensity of 5.2 mW/cm^2^ using a 670 nm laser source (n = 7, as multiple experiments, ** *p* < 0.01 compared to free Ce6). A detailed description of each sample was given in the Figure 3 legend.

To evaluate the in vitro phototoxicity of dHA samples, four types of cell were cultured with or without tumor-specific receptors, such as CD44 receptor and integrin α_v_β_3_ [MDA-MB-231 cells (CD44+, integrin α_v_β_3_+), A549 cells (CD44+, integrin α_v_β_3_−), BT-474 cells (CD44−, integrin α_v_β_3_+), and NIH3T3 cells (CD44−, integrin α_v_β_3_−)] [4,11,50,55] using a cell-culture medium containing type I collagen solution with or without hydroxyapatite particles (Figure 4) and performed in vitro phototoxicity test of each sample [46]. In addition, before this test, the collagen solution in the cell-culture medium was solidified (resulting in producing collagen gel), and the obtained gel was flipped to expose the cells on the surface containing hydroxyapatite particles (mimicking in vivo bone environment). The cells growing on collagen gel with hydroxyapatite particles were treated with dHA samples (equivalent Ce6 10 μg/mL), (ALN/cRGD)@HDOC-Ce6 NP (equivalent Ce6 10 μg/mL), and free Ce6 (10 μg/mL) for 4 h, irradiated using a 670 nm laser source (5.2 mW/cm^2^ for 10 min), and then further incubated for 12 h. Subsequently, cell viability was evaluated using a CCK-8 assay. As shown in Figure 4a, (ALN/cRGD)@dHA-Ce6 exhibited highly increased MDA-MB-231 cell death, probably owing to their efficient binding ability to CD44 receptors, integrin α_v_β_3_, and hydroxyapatite particles because of the presence of HA, cRGD, and ALN moieties [4,25,26,27,28,29,30,31,32,33,34,35]. However, the (ALN/cRGD)@HDOC-Ce6 NP resulted in relatively low phototoxicity to MDA-MB-231 cells because of their reduced cellular uptakes, probably owing to their relatively large particle size. Furthermore, the dHA-Ce6, (ALN)@dHA-Ce6, and (cRGD)@dHA-Ce6 showed less effectiveness in MDA-MB-231 cell death because of their absence of ALN moieties, cRGD moieties, or both. Similarly, (ALN/cRGD)@dHA-Ce6 exhibited no significant phototoxicity to A549 cells without integrin α_v_β_3_, BT-474 cells without CD44 receptors, and NIH3T3 cells without CD44 receptors and integrin α_v_β_3_ (Figure 4a). In addition, compared with the results of dHA-Ce6 and (cRGD)@dHA-Ce6, (ALN/cRGD)@dHA-Ce6 and (ALN)@dHA-Ce6 showed relatively increased cytotoxicity to A549 cells with CD44 receptors, indicating that the binding ability of ALN moieties to hydroxyapatite particles helps to enhance cell cytotoxicity. As a result, the cell viability of MDA-MB-231 cells treated with (ALN/cRGD)@dHA-Ce6 was approximately 2.1, 1.7, 1.5, 2.6, and 1.9 times smaller than those of MDA-MB-231 cells treated with the (ALN/cRGD)@HDOC-Ce6 NP, (cRGD)@dHA-Ce6, (ALN)@dHA-Ce6, dHA-Ce6, and free Ce6, respectively.

Figure 4b shows that the (ALN)@dHA, (ALN/cRGD)@HDOC-Ce6 NP, and (ALN/cRGD)@dHA-Ce6 showed relatively decreased cell phototoxicity under the modified experimental condition without hydroxyapatite particles that can be binding with ALN moieties. These results indicate that the multiligand targeting ability of (ALN/cRGD)@dHA-Ce6 with HA, cRGD, and ALN could significantly improve cell death of bone-metastasized MDA-MB-231 tumor cells. In addition, each dHA sample and (ALN/cRGD)@HDOC-Ce6 NP without light irradiation showed negligible cytotoxicity up to 200 μg/mL during co-incubation with MDA-MB-231, A549, BT-474, and NIH3T3 cells (Figure S5), indicating their negligible original toxicity.

### 3.5. In Vitro/In Vivo Evaluation of dHA Samples

**Figure 5:**
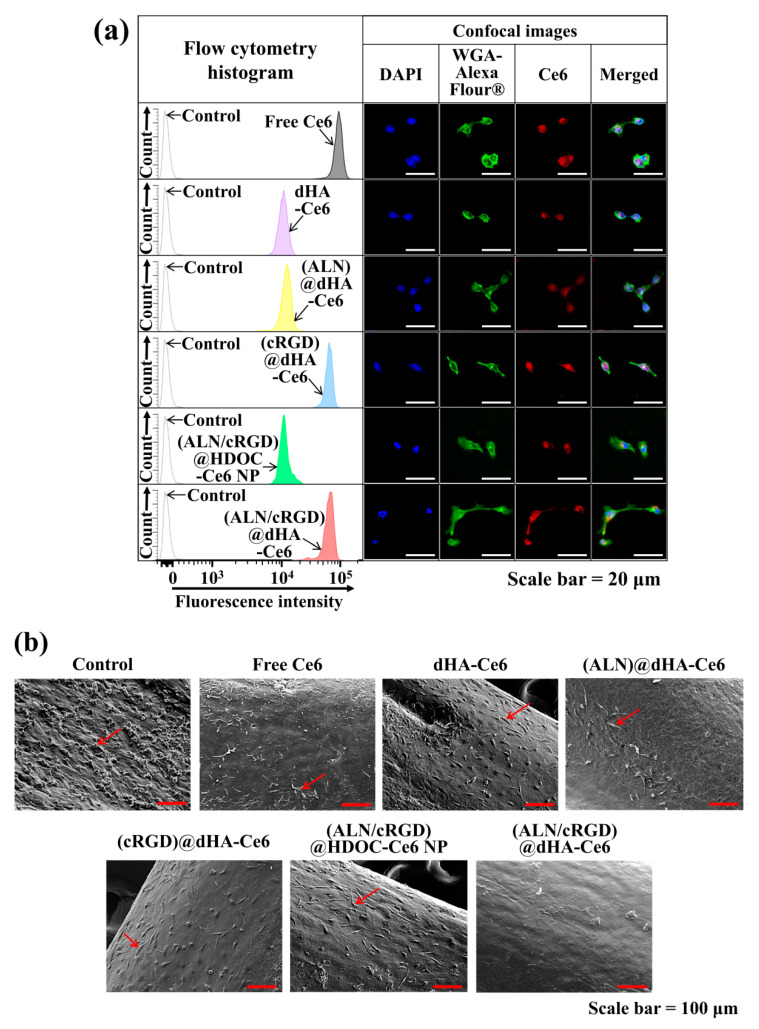
(**a**) Flow cytometry analysis and confocal images of MDA-MB-231 cells treated with free Ce6 (10 μg/mL) or each sample (equivalent Ce6 10 μg/mL) at 37 °C for 4 h. (**b**) SEM images of MDA-MB-231 cells (cultured on tibia of ex vivo bone metastasized model) treated free Ce6 (10 μg/mL) or each sample (equivalent Ce6 10 μg/mL). Red arrows indicate tumor cells. A detailed description of each sample was given in the Figure 3 legend.

To verify the cellular uptake behaviors of dHA samples to MDA-MB-231 tumor cells, we performed in vitro cell tests using a FACSCalibur^TM^ flow cytometer and a confocal laser scanning microscope [4,14,41,42,56]. As shown in Figure 5a, the extremely small-sized (ALN/cRGD)@dHA-Ce6 and (cRGD)@dHA-Ce6 exhibited highly increased Ce6 fluorescence intensity in MDA-MB-231 cells, probably owing to their highly efficient binding ability to CD44 receptors and integrin α_v_β_3_ in MDA-MB-231 cells during 4 h incubation [28,29,30,31,32,33,34,35]. However, (ALN/cRGD)@HDOC-Ce6 NP (≈100 nm in diameter) showed relatively reduced cellular uptakes, probably because of their relatively delayed absorption. In addition, dHA-Ce6 and (ALN)@dHA-Ce6 showed relatively decreased cellular uptakes owing to the absence of cRGD ligands that can bind to the integrin α_v_β_3_ of MDA-MB-231 cells.

Next, MDA-MB-231 tumor cells were cultured on the surface of ex vivo tibias (as an ex vivo bone metastasized tumor model) to determine the phototoxicity of the dHA samples under ex vivo conditions [27,47]. After 48 h, the cells growing on tibias were treated with dHA samples (equivalent Ce6 10 μg/mL), (ALN/cRGD)@HDOC-Ce6 NP (equivalent Ce6 10 μg/mL), and free Ce6 (10 μg/mL) for 4 h, irradiated using a 670 nm laser source (5.2 mW/cm^2^ for 10 min), and then further incubated for 12 h. Figure 5b shows the SEM images of tibias treated with each sample. In particular, the tibia treated with (ALN/cRGD)@dHA-Ce6 exhibited a decreased population of tumor cells, indicating highly increased tumor ablation ability of (ALN/cRGD)@dHA-Ce6. However, the tibia treated with the other samples showed partial proliferation and distribution of tumor cells on their surface, revealing their limited therapeutic activities.

**Figure 6:**
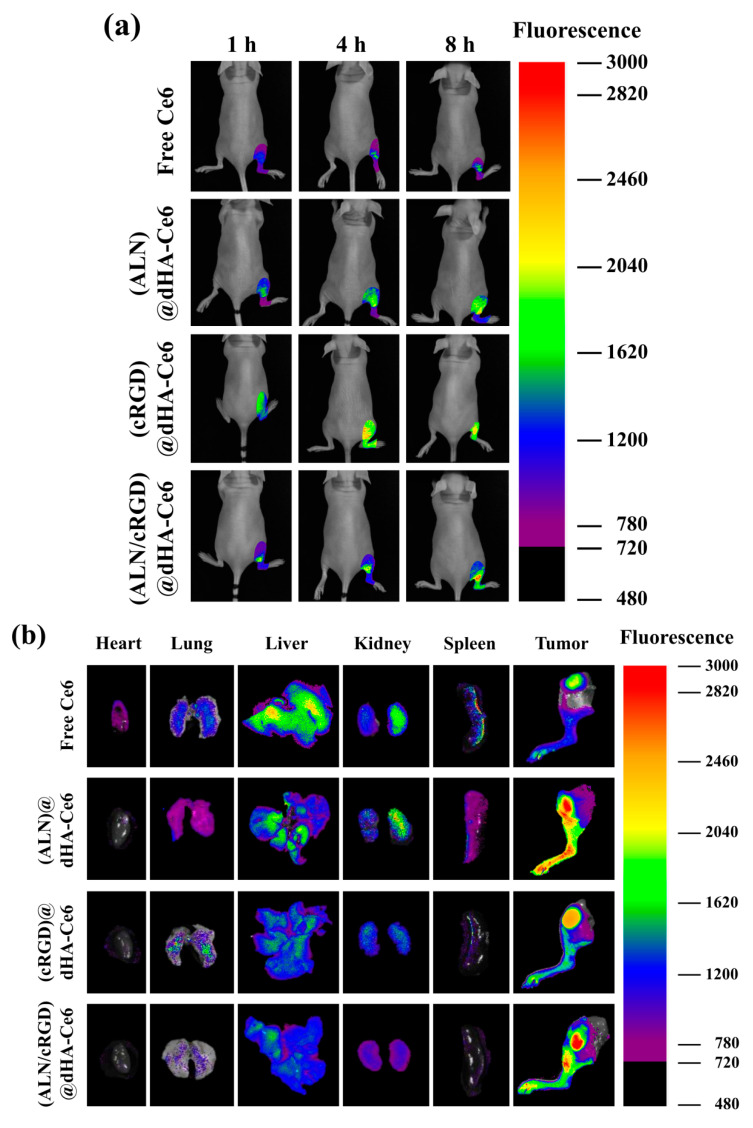

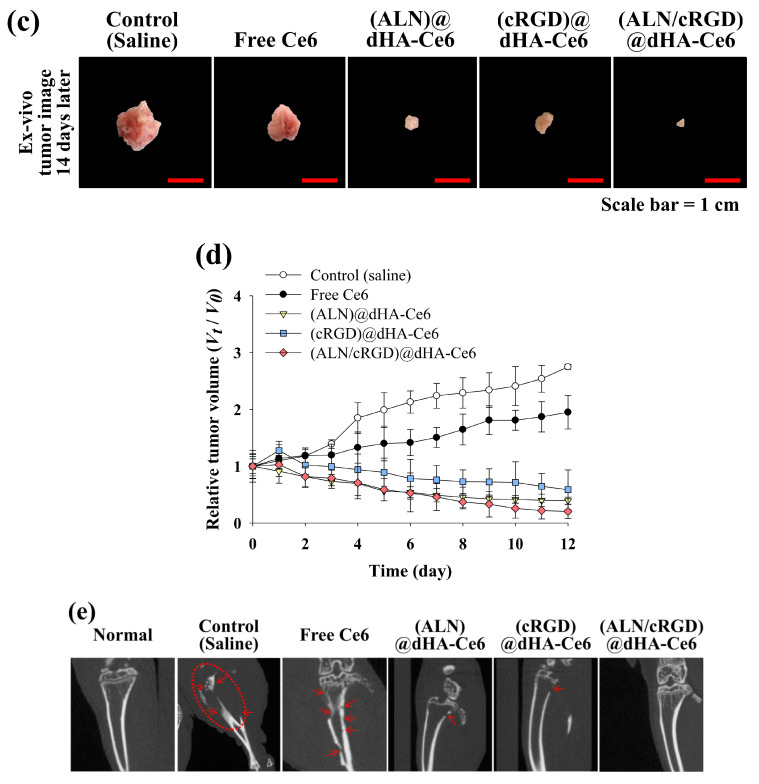
(**a**) In vivo noninvasive photoluminescent tumor imaging of free Ce6 (2.5 mg/kg) or each sample (equivalent Ce6 2.5 mg/kg) intravenously injected into MDA-MB-231 tumor-bearing nude mice. Fluorescent tumor images were obtained at 1, 4, and 8 h post-injection. (**b**) Ex vivo fluorescence images of major organs and tumors at 24 h post-injection. (**c**) Optical images of ex vivo tumors extracted from MDA-MB-231 tumor-bearing nude mice at 14 days post-injection of saline, free Ce6 (2.5 mg/kg), or each sample (equivalent Ce6 2.5 mg/kg). (**d**) Relative tumor volume change (*V_t_*/*V_0_*, where *V_t_* is the tumor volume at a given time and *V_0_* is the initial tumor volume) of MDA-MB-231 tumor-bearing nude mice treated with saline, free Ce6 (2.5 mg/kg) or each sample (equivalent Ce6 2.5 mg/kg). (**e**) In vivo X-ray CT images of MDA-MB-231 tumor-bearing nude mice treated with control (saline), free Ce6 (2.5 mg/kg), or each sample (equivalent Ce6 2.5 mg/kg). The tumor site is indicated by a dashed circle and arrow.

The in vivo tumor-targeting ability of each dHA sample was also investigated using a tumor-bearing mouse model with MDA-MB-231 tumors implanted on the right tibia. The dHA sample (equivalent Ce6 2.5 mg/kg) or free Ce6 (2.5 mg/kg) was intravenously injected to the tail vein of mice, and their fluorescence images were obtained for 24 h using a FOBI (Figure 6a,b) [4,49]. As shown in Figure 6a, (ALN/cRGD)@dHA-Ce6 exhibited a strong Ce6 fluorescence signal at the tumor sites after 8 h post-injection. However, (ALN)@dHA-Ce6, (cRGD)@dHA-Ce6, and free Ce6 exhibited relatively weak Ce6 fluorescence because of the relatively weak tumor-binding ability. Furthermore, we verified the Ce6 fluorescence intensity in excised organs (heart, lung, liver, kidney, spleen, and tumor) at 24 h post-injection (Figure 6b) [4,49]. As a result, (ALN/cRGD)@dHA-Ce6 and (ALN)@dHA-Ce6 exhibited strong Ce6 fluorescence in the tumor site on the right tibia. In contrast, (cRGD)@dHA-Ce6 without ALN and free Ce6 exhibited relatively weak Ce6 fluorescence in the tumor site. It was observed that the fluorescence intensity of (ALN/cRGD)@dHA-Ce6 in normal organs (heart, lung, liver, kidney, and spleen) was relatively weak, indicating reduced accumulation to normal organs.

To confirm the in vivo photodynamic antitumor efficacy of the dHA samples, the dHA samples (equivalent Ce6 2.5 mg/kg), free Ce6 (2.5 mg/kg), and control (saline) were intravenously injected to MDA-MB-231 tumor-bearing nude mice, and tumor sites were irradiated using a 670 nm laser source (5.2 mW/cm^2^ for 10 min) (Figure 6c). Importantly, the (ALN/cRGD)@dHA-Ce6 resulted in a significant tumor volume regression after 14 days post-injection (Figure 6d); the relative tumor volume in the nude mice injected with the (ALN/cRGD)@dHA-Ce6 was approximately 2.9, 1.9, 9.5, and 13.4 times smaller than those of the nude mice injected with the (cRGD)@dHA-Ce6, (ALN)@dHA-Ce6, free Ce6, and saline (control), respectively. Furthermore, the micro CT images (Figure 6e) support that (ALN/cRGD)@dHA-Ce6 was highly effective in inhibiting MDA-MB-231 tumor cells, presenting no difference from the CT image of a normal right leg.

Overall, the results of in vitro/in vivo studies demonstrate that (ALN/cRGD)@dHA-Ce6-mediated photodynamic antitumor therapy can provide an efficient strategy to treat metastatic bone tumors. Furthermore, we confirmed that the targeting efficiency of ALN moieties to bone-metastasized tumors was quite high. Nevertheless, since the in vivo antitumor activity of (cRGD)@dHA-Ce6 with cRGD moieties is not bad, we think that (ALN/cRGD)@dHA-Ce6 with multiple tumor-targeting ability will have various advantages for targeting complicated in vivo tumors. Of course, more clear proof should be clarified through various in vivo antitumor experiments in the future.

## 4. Conclusions

In this study, (ALN/cRGD)@dHA-Ce6 was successfully synthesized for highly efficient photodynamic therapy of metastatic tumors. The in vitro/in vivo results demonstrated that the multiple tumor-targeting ability of (ALN/cRGD)@dHA-Ce6 with HA, cRGD, and ALN as tumor-binding ligands enhanced tumor cell binding affinity and improved photodynamic tumor ablation. Based on the results of this study, it is concluded that the extremely small-sized dot system with multiple ligands can be effective in treating metastatic tumors, although in vivo pharmacokinetics evaluation should be performed in the future.

## Supplementary Materials

The following are available online at https://www.mdpi.com/2227-9059/8/11/492/s1, Figure S1: ^1^H-NMR peaks of (ALN/cRGD)@dHA-Ce6. Figure S2: ^1^H-NMR peaks of (ALN/cRGD)@HDOC-Ce6. Figure S3: ^1^H-NMR peaks of (ALN)@dHA-Ce6. Figure S4: ^1^H-NMR peaks of (cRGD)@dHA-Ce6. Figure S5: In vitro cell viability were determined by a CCK-8 assay of (a) MDA-MB-231, (b) A549, (c) BT-474, and (d) NIH3T3 cells treated with dHA-Ce6, (ALN)@dHA-Ce6, (cRGD)@dHA-Ce6, (ALN/cRGD)@HDOC-Ce6 NP and (ALN/cRGD)@dHA-Ce6. Figure S6: The emission spectra (at λ_ex_ of 400 nm and λ_em_ of 600–750 nm) of free Ce6 (10 μg/mL) and each sample (equivalent Ce6 10 μg/mL) in PBS (pH 7.4, 150 mM), which is reconstructed by separating the results of Figure 2e.

## References

[1] Jung Y.K., Shin E., Kim B. (2015). Cell nucleus-targeting zwitterionic carbon dots. Sci. Rep..

[2] Dong Y., Fang Q., Wu H., Wan L., Lin Y., Lu C.H., Chi Y., Yang H.H. (2016). Fullerene-structural carbon-based dots from C_60_ molecules and their optical properties. Part. Part. Syst. Charact..

[3] Molaei M.J. (2019). Carbon quantum dots and their biomedical and therapeutic applications: A review. RSC Adv..

[4] Noh G.J., Oh K.T., Youn Y.S., Lee E.S. (2020). Cyclic RGD-conjugated hyaluronate dot bearing cleavable doxorubicin for multivalent tumor targeting. Biomacromolecules.

[5] Choi H.S., Liu W., Liu F., Nasr K., Misra P., Bawendi M.G., Frangioni J.V. (2010). Design considerations for tumour-targeted nanoparticles. Nat. Nanotechnol..

[6] Choi Y., Kim K., Hong S., Kim H., Kwon Y.J., Song R. (2011). Intracellular protein target detection by quantum dots optimized for live cell imaging. Bioconjug. Chem..

[7] Chang J.C., Kovtun O., Blakely R.D., Rosenthal S.J. (2012). Labeling of neuronal receptors and transporters with quantum dots. Wiley Interdiscip. Rev. Nanomed. Nanobiotechnol..

[8] Karakoti A.S., Shukla R., Shanker R., Singh S. (2015). Surface functionalization of quantum dots for biological applications. Adv. Colloid Interface Sci..

[9] Elsabahy M., Wooley K.L. (2013). Cytokines as biomarkers of nanoparticle immunotoxicity. Chem. Soc. Rev..

[10] Havrdova M., Hola K., Skopalik J., Tomankova K., Petr M., Cepe K., Polakova K., Tucek J., Bourlinos A., Zboril R. (2016). Toxicity of carbon dots—Effect of surface functionalization on the cell viability, reactive oxygen species generation and cell cycle. Carbon.

[11] Choi E.J., Park H., Noh G.J., Lee E.S. (2019). Tumor cell-on fluorescence imaging agent using hyaluronate dots. Carbohydr. Polym..

[12] Bagalkot V., Zhang L., Levy-Nissenbaum E., Jon S., Kantoff P.W., Langer R., Farokhzad O.C. (2007). Quantum dot-aptamer conjugates for synchronous cancer imaging, therapy, and sensing of drug delivery based on bi-fluorescence resonance energy transfer. Nano Lett..

[13] Kulkarni N.S., Guererro Y., Gupta N., Muth A., Gupta V. (2019). Exploring potential of quantum dots as dual modality for cancer therapy and diagnosis. J. Drug Deliv. Sci. Technol..

[14] Choi E.J., Lee J.M., Youn Y.S., Na K., Lee E.S. (2018). Hyaluronate dots for highly efficient photodynamic therapy. Carbohydr. Polym..

[15] Pourtau L., Oliveira H., Thevenot J., Wan Y.L., Brisson A.R., Sandre O., Miraux S., Thiaudiere E., Lecommandoux S. (2013). Antibody-functionalized magnetic polymersomes: In vivo targeting and imaging of bone metastases using high resolution MRI. Adv. Healthc. Mater..

[16] Bersini S., Jeon J.S., Dubini G., Arrigoni C., Chung S., Charest J.L., Moretti M., Kamm R.D. (2014). A microfluidic 3D in vitro model for specificity of breast cancer metastasis to bone. Biomaterials.

[17] Salamanna F., Contartese D., Maglio M., Fini M. (2016). A systematic review on in vitro 3D bone metastases models. A new horizon to recapitulate the native clinical scenario?. Oncotarget.

[18] Adjei I.M., Temples M.N., Brown S.B., Sharma B. (2018). Targeted nanomedicine to treat bone metastasis. Pharmaceutics.

[19] Zhang X.H.F., Wang Q., Gerald W., Hudis C.A., Norton L., Smid M., Foekens J.A., Massagué J. (2009). Latent bone metastasis in breast cancer tied to Src-dependent survival signals. Cancer Cell.

[20] Wang F., Chen L., Zhang R., Chen Z., Zhu L. (2014). RGD peptide conjugated liposomal drug delivery system for enhance therapeutic efficacy in treating bone metastasis from prostate cancer. J. Control. Release.

[21] Vinay R., KusumDevi V. (2016). Potential of targeted drug delivery system for the treatment of bone metastasis. Drug Deliv..

[22] Dang L., Liu J., Li F., Wang L., Li D., Guo B., He X., Jiang F., Liang C., Liu B. (2016). Targeted delivery systems for molecular therapy in skeletal disorders. Int. J. Mol. Sci..

[23] Lin J.H. (1996). Bisphosphonates: A review of their pharmacokinetic properties. Bone.

[24] Chaudhari K.R., Kumar A., Khandelwal V.K.M., Ukawala M., Manjappa A.S., Mishra A.K., Monkkonen J., Murthy R.S.R. (2012). Bone metastasis targeting: A novel approach to reach bone using zoledronate anchored PLGA nanoparticle as carrier system loaded with docetaxel. J. Control. Release.

[25] Farrell K.B., Karpeisky A., Thamm D.H., Zinnen S. (2018). Bisphosphonate conjugation for bone specific drug targeting. Bone Rep..

[26] Rotman S.G., Thompson K., Grijpma D.W., Richards R.G., Moriarty T.F., Eglin D., Guillaume O. (2019). Development of bone seeker–functionalised microspheres as a targeted local antibiotic delivery system for bone infections. J. Orthop. Transl..

[27] Bai S.B., Cheng Y., Liu D.Z., Ji Q.F., Liu M., Zhang B.L., Mei Q.B., Zhou S.Y. (2020). Bone-targeted PAMAM nanoparticle to treat bone metastases of lung cancer. Nanomedicine.

[28] Culty M., Miyake K., Kincade P.W., Sikorski E., Butcher E.C., Underhill C., Silorski E. (1990). The hyaluronate receptor is a member of the CD44 (H-CAM) family of cell surface glycoproteins. J. Cell Biol..

[29] Park S., Oh K.T., Kwag D.S., Lee U.Y., Lee D.J., Lee E.S. (2013). Photoresponsive hyaluronate nanogel as an anticancer drug carrier. Polym. Adv. Technol..

[30] Arabi L., Badiee A., Mosaffa F., Jaafari M.R. (2015). Targeting CD44 expressing cancer cells with anti-CD44 monoclonal antibody improves cellular uptake and antitumor efficacy of liposomal doxorubicin. J. Control. Release.

[31] Cortes-Dericks L., Schmid R.A. (2017). CD44 and its ligand hyaluronan as potential biomarkers in malignant pleural mesothelioma: Evidence and perspectives. Respir. Res..

[32] Lee J.M., Park H., Oh K.T., Lee E.S. (2018). pH-Responsive hyaluronated liposomes for docetaxel delivery. Int. J. Pharm..

[33] Zhao Y., Bachelier R., Treilleux I., Pujuguet P., Peyruchaud O., Baron R., Clément-Lacroix P., Clézardin P. (2007). Tumor α_v_β_3_ integrin is a therapeutic target for breast cancer bone metastases. Cancer Res..

[34] Dal Pozzo A., Esposito E., Ni M., Muzi L., Pisano C., Bucci F., Vesci L., Castorina M., Penco S. (2010). Conjugates of a novel 7-substituted camptothecin with RGD-peptides as α_v_β_3_ integrin ligands: An approach to tumor-targeted therapy. Bioconjug. Chem..

[35] Kim S.K., Lee J.M., Oh K.T., Lee E.S. (2017). Extremely small-sized globular poly(ethylene glycol)-cyclic RGD conjugates targeting integrin α_v_β_3_ in tumor cells. Int. J. Pharm..

[36] Kwag D.S., Park K., Oh K.T., Lee E.S. (2013). Hyaluronated fullerenes with photoluminescent and antitumoral activity. Chem. Commun..

[37] Lee H., Park H., Noh G.J., Lee E.S. (2018). pH-responsive hyaluronate-anchored extracellular vesicles to promote tumor-targeted drug delivery. Carbohydr. Polym..

[38] Park J., Lee H., Youn Y.S., Oh K.T., Lee E.S. (2020). Tumor-homing pH-sensitive extracellular vesicles for targeting heterogeneous tumors. Pharmaceutics.

[39] Kim S., Park J., Youn Y.S., Oh K.T., Bae J.H., Lee E.S. (2015). Hoechst 33258–conjugated hyaluronated fullerene for efficient photodynamic tumor therapy and necrotic tumor targeting. J. Bioact. Compat. Polym..

[40] Kim S.W., Oh K.T., Youn Y.S., Lee E.S. (2014). Hyaluronated nanoparticles with pH-and enzyme-responsive drug release properties. Colloids Surf. B Biointerfaces.

[41] Koo M., Oh K.T., Noh G., Lee E.S. (2018). Gold nanoparticles bearing a tumor pH-sensitive cyclodextrin cap. ACS Appl. Mater. Interfaces.

[42] Yu H.S., Park H., Tran T.H., Hwang S.Y., Na K., Lee E.S., Oh K.T., Oh D.X., Park J. (2019). Poisonous caterpillar-inspired chitosan nanofiber enabling dual photothermal and photodynamic tumor ablation. Pharmaceutics.

[43] Oh N.M., Kwag D.S., Oh K.T., Youn Y.S., Lee E.S. (2012). Electrostatic charge conversion processes in engineered tumor-identifying polypeptides for targeted chemotherapy. Biomaterials.

[44] Lee U.Y., Youn Y.S., Park J., Lee E.S. (2014). Y-Shaped ligand-driven gold nanoparticles for highly efficient tumoral uptake and photothermal ablation. ACS Nano.

[45] Lee D.J., Youn Y.S., Lee E.S. (2015). Photodynamic tumor therapy of nanoparticles with chlorin e6 sown in poly(ethylene glycol) forester. J. Mater. Chem. B.

[46] Pham T.T., Nguyen H.T., Phung C.D., Pathak S., Regmi S., Ha D.H., Kim J.O., Yong C.S., Kim S.K., Choi J.E. (2019). Targeted delivery of doxorubicin for the treatment of bone metastasis from breast cancer using alendronate-functionalized graphene oxide nanosheets. J. Ind. Eng. Chem..

[47] Cooper C.R., McLean L., Walsh M., Taylor J., Hayasaka S., Bhatia J., Pienta K.J. (2000). Preferential adhesion of prostate cancer cells to bone is mediated by binding to bone marrow endothelial cells as compared to extracellular matrix components in vitro. Clin. Cancer Res..

[48] Sun W., Ge K., Jin Y., Han Y., Zhang H., Zhou G., Yang X., Liu D., Liu H., Liang X.J. (2019). Bone-targeted nanoplatform combining zoledronate and photothermal therapy to treat breast cancer bone metastasis. ACS Nano.

[49] Kim D.H., Im B.N., Hwang H.S., Na K. (2018). Gemcitabine-loaded DSPE-PEG-PheoA liposome as a photomediated immune modulator for cholangiocarcinoma treatment. Biomaterials.

[50] Yamada Y., Hashida M., Harashima H. (2015). Hyaluronic acid controls the uptake pathway and intracellular trafficking of an octaarginine-modified gene vector in CD44 positive- and CD44 negative-cells. Biomaterials.

[51] Zhan J., Wang L., Liu S., Chen J., Ren L., Wang Y. (2015). Antimicrobial hyaluronic acid/poly (amidoamine) dendrimer multilayer on poly (3-hydroxybutyrate-co-4-hydroxybutyrate) prepared by a layer-by-layer self-assembly method. ACS Appl. Mater. Interfaces.

[52] Ke J., Dou H., Zhang X., Uhagaze D.S., Ding X., Dong Y. (2016). Determination of pKa values of alendronate sodium in aqueous solution by piecewise linear regression based on acid-base potentiometric titration. J. Pharm. Anal..

[53] Mekhail G.M., Kamel A.O., Awad G.A., Mortada N.D., Rodrigo R.L., Spagnuolo P.A., Wettig S.D. (2016). Synthesis and evaluation of alendronate-modified gelatin biopolymer as a novel osteotropic nanocarrier for gene therapy. Nanomedicine.

[54] Park S.Y., Baik H.J., Oh Y.T., Oh K.T., Youn Y.S., Lee E.S. (2011). A smart polysaccharide/drug conjugate for photodynamic therapy. Angew. Chem. Int. Ed..

[55] Mashayekhi V., Op’t Hoog C., Oliveira S. (2019). Vascular targeted photodynamic therapy: A review of the efforts towards molecular targeting of tumor vasculature. J. Porphyr. Phthalocyanines.

[56] Cho M.H., Li Y., Lo P., Lee H., Choi Y. (2020). Fucoidan-based theranostic nanogel for enhancing imaging and photodynamic therapy of cancer. Nano Micro Lett..

