# Supplementary material for: Alendronate/cRGD-Decorated Ultrafine Hyaluronate Dot Targeting Bone Metastasis
Source: Biomedicines. 2020 Nov 11;8(11):492. doi: 10.3390/biomedicines8110492 (PMC7696888; doi:10.3390/biomedicines8110492)
Supplement: Supplementary file 1 [file biomedicines-08-00492-s001.pdf]

biomedicines

MDPI

Supplementary Materials

Alendronate/cRGD-decorated ultrafine hyaluronate dot targeting bone metastasis

Eunsol Lee [1,†], Jaeduk Park [1,†], Yu Seok Youn [2], Kyung Taek Oh [3], Dongin Kim [4] and Eun Seong Lee [1,5,*]

[1] Department of Biotechnology, The Catholic University of Korea, 43 Jibong-ro, Bucheon-si, Gyeonggi-do 14662, Republic of Korea; eunsollee13@gmail.com (E.L.); jduck0309@naver.com (J.P.); eslee@catholic.ac.kr (E.S.L.)

[2] School of Pharmacy, SungKyunKwan University, 2066 Seobu-ro, Jangan-gu, Suwon, Gyeonggi-do 16419, Republic of Korea; ysyoun@skku.edu (Y.S.Y.)

[3] College of Pharmacy, Chung-Ang University, 221 Heukseok dong, Dongjak-gu, Seoul 06974, Republic of Korea; kyungoh@cau.ac.kr (K.T.O.)

[4] Department of Pharmaceutical Sciences, College of Pharmacy, University of Oklahoma Health Sciences Center, 1110 N Stonewall Ave, Oklahoma City, OK 73117, USA; Dongin-Kim@ouhsc.edu (D.K)

[5] Department of Biomedical Chemical Engineering, The Catholic University of Korea, 43 Jibong-ro, Bucheon-si, Gyeonggi-do 14662, Republic of Korea

* Correspondence: eslee@catholic.ac.kr; Tel.: +82-02-2164-4921

† These authors equally contributed to this work.

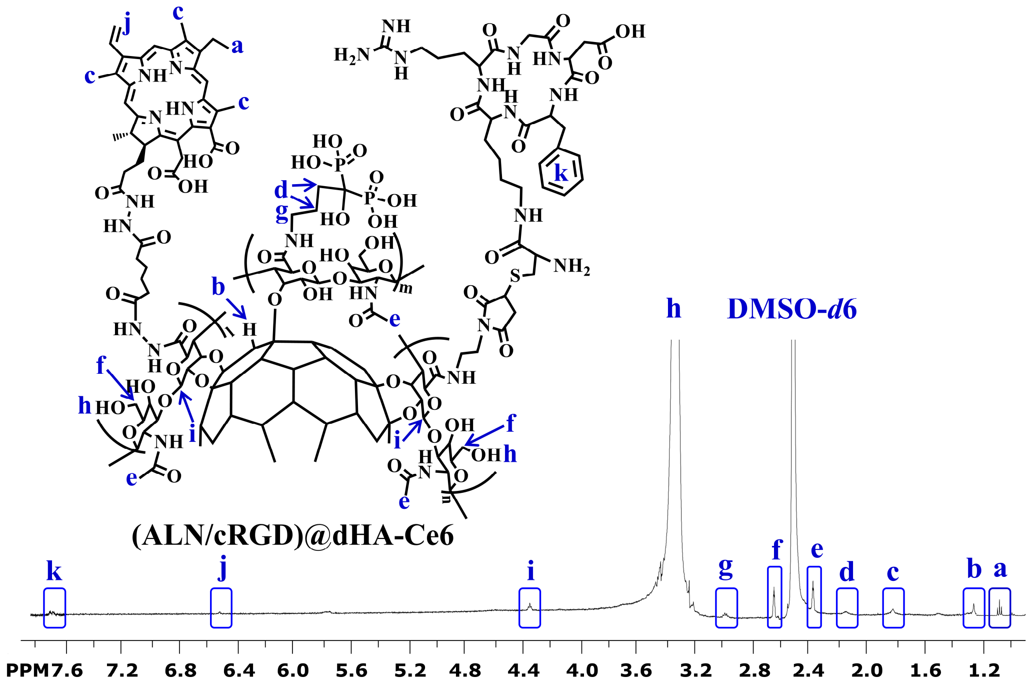

Figure S1. ^{1}H-NMR peaks of (ALN/cRGD)@dHA-Ce6.

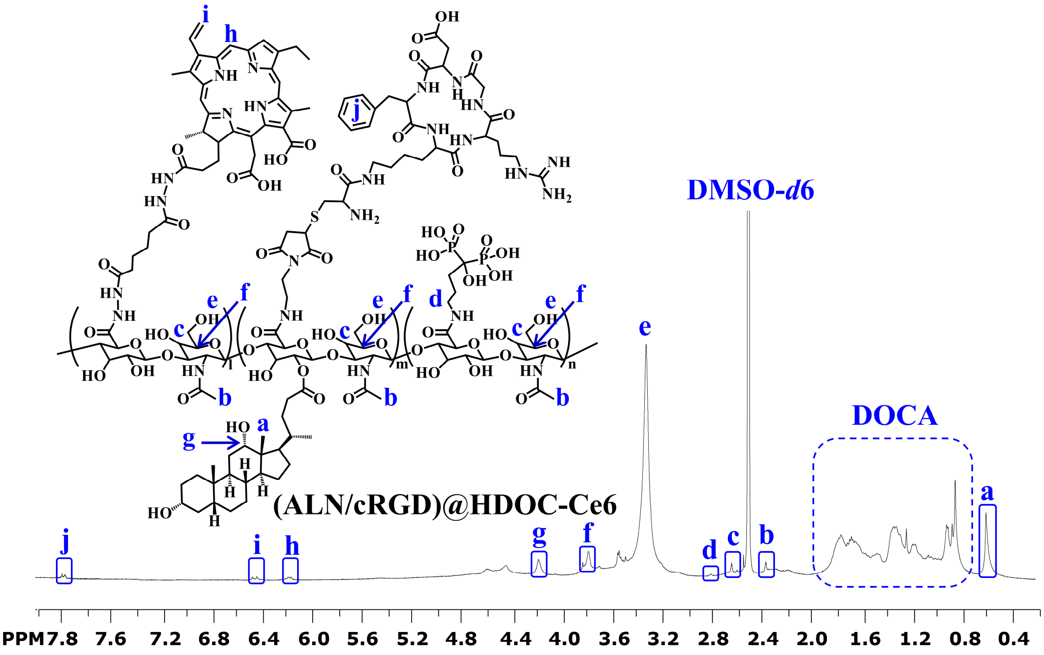

Figure S2. ^{1}H-NMR peaks of (ALN/cRGD)@HDOC-Ce6.

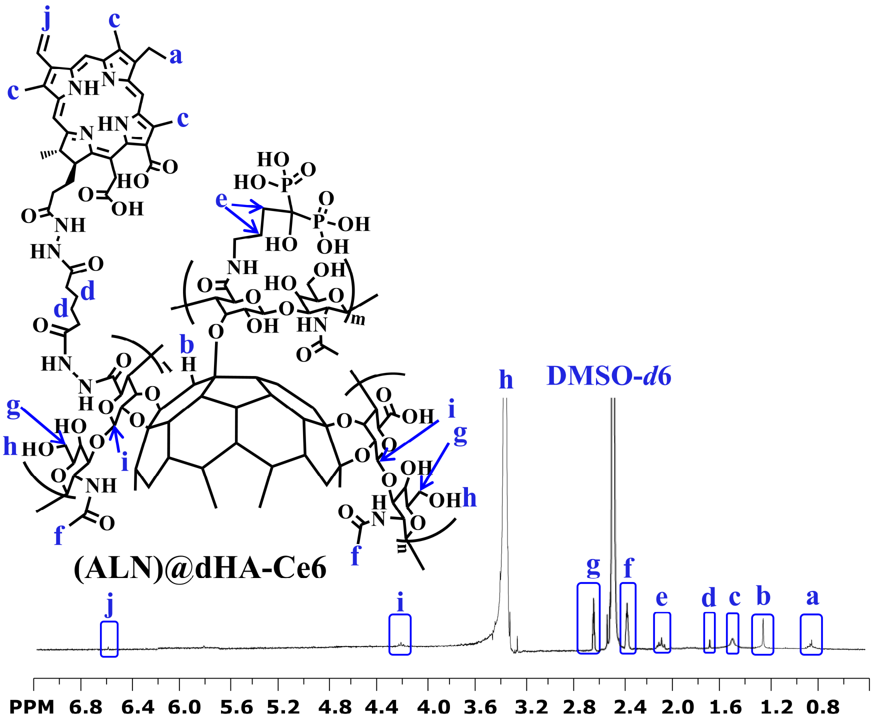

Figure S3. ^{1}H-NMR peaks of (ALN)@dHA-Ce6.

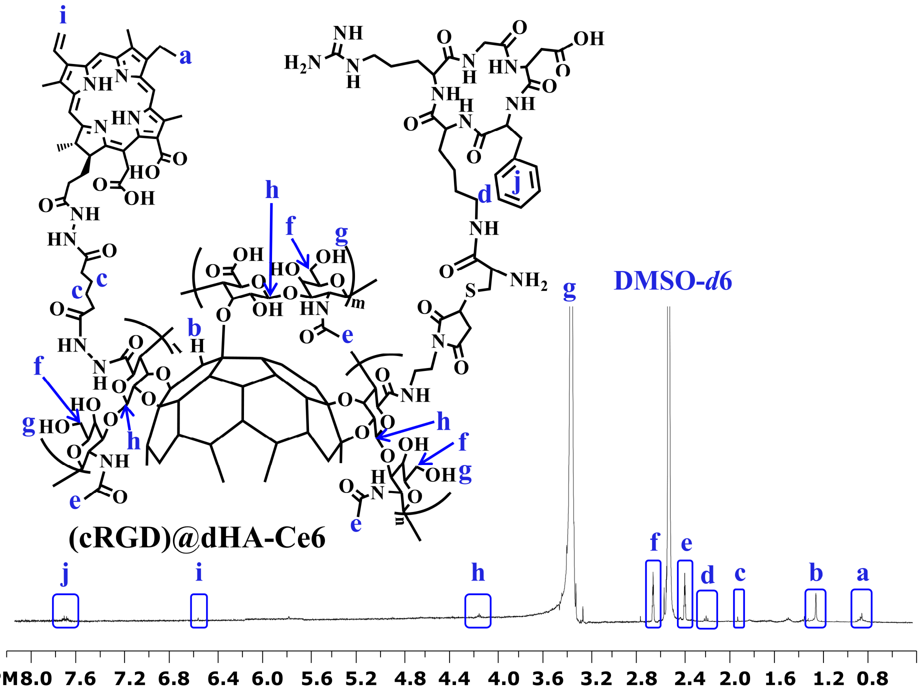

Figure S4. ^{1}H-NMR peaks of (cRGD)@dHA-Ce6.

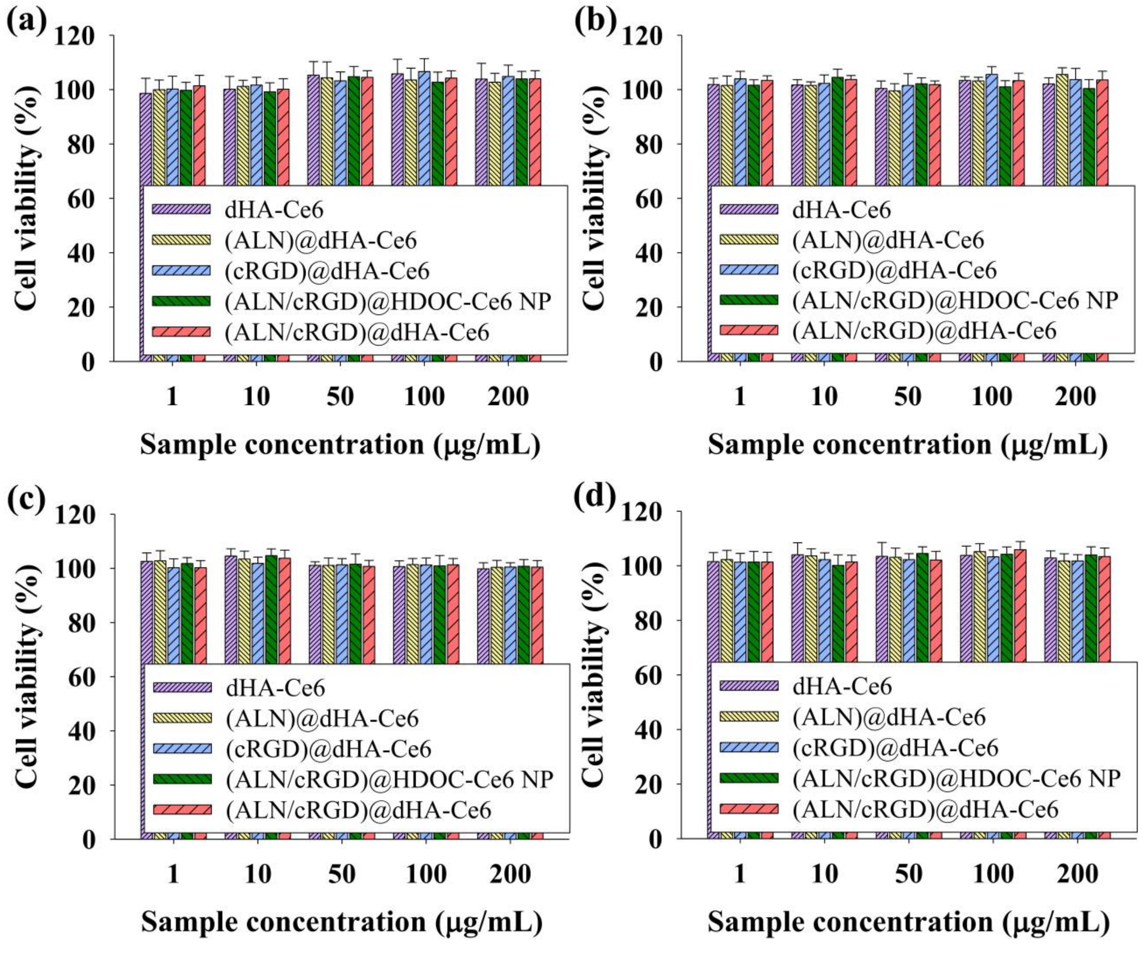

Figure 5. *In vitro* cell viability was determined by a CCK-8 assay of (a) MDA-MB-231, (b) A549, (c) BT-474, and (d) NIH3T3 cells treated with dHA-Ce6, (ALN)@dHA-Ce6, (cRGD)@dHA-Ce6, (ALN/cRGD)@HDOC-Ce6 NP and (ALN/cRGD)@dHA-Ce6 (n = 7, as multiple experiments).

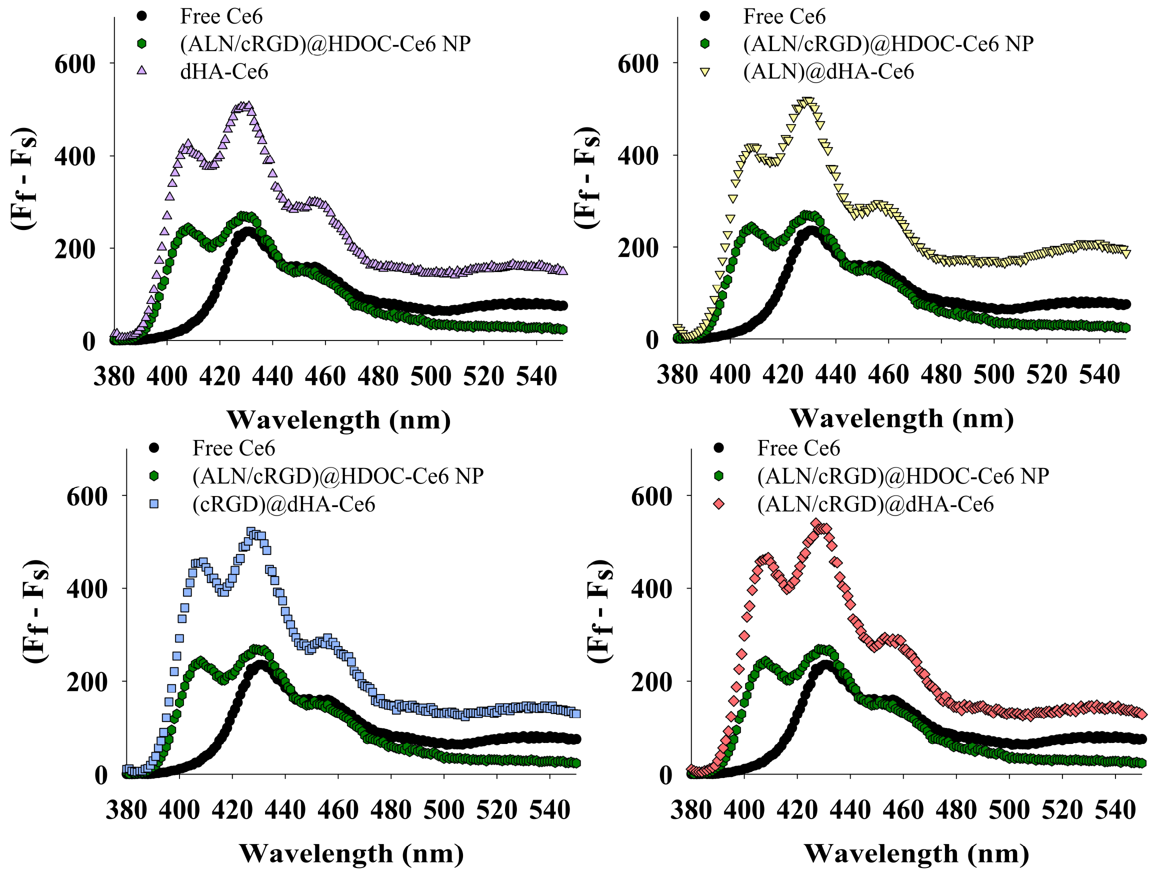

Figure 6. The emission spectra (at λ_{ex} of 400 nm and λ_{em} of 600-750 nm) of free Ce6 (10 μg/mL) and each sample (equivalent Ce6 10 μg/mL) in PBS (pH 7.4, 150 mM). Which is reconstructed by separating the results of Figure 2e. The 9,10-dimethylanthracene (DMAT) fluorescence change (at λ_{ex} of 360 nm and λ_{em} of 380-550 nm) of free Ce6 (10 μg/mL) and each sample (equivalent Ce6 10 μg/mL) in PBS (pH 7.4, 150 mM). The singlet oxygen generation is indicated by the DMAT fluorescence intensity change (F_f - F_s, where F_f is the fluorescence intensity of DMAT and F_s is the fluorescence intensity of DMAT mixed with the sample).
